# Combination of genomic instability score and *TP53* status for prognosis prediction in lung adenocarcinoma

**DOI:** 10.1038/s41698-023-00465-x

**Published:** 2023-10-31

**Authors:** Juan Feng, Yang Lan, Feng Liu, Ye Yuan, Jia Ge, Sen Wei, Hu Luo, Jianjun Li, Tao Luo, Xiuwu Bian

**Affiliations:** 1 Institute of Pathology and Southwest Cancer Center, Southwest Hospital, Third Military Medical University (Army Medical University) and Key Laboratory of Tumor Immunopathology, Ministry of Education of China, 400038 Chongqing, China; 2 Department of Respiratory and Critical Care Medicine, Southwest Hospital, Third Military Medical University (Army Medical University), 400038 Chongqing, China; 3 Department of Oncology, Southwest Hospital, Third Military Medical University (Army Medical University), 400038 Chongqing, China

**Keywords:** Tumour biomarkers, Non-small-cell lung cancer, Molecular medicine, Risk factors

## Abstract

The genomic instability (GI) /homologous recombination deficiency (HRD) score, calculated as the sum of the events of loss of heterozygosity (LOH), large-scale state transition (LST) and telomere allele imbalance (TAI), is used to guide the choice of treatment in several cancers, but its relationship with genomic features, clinicopathological characteristics and prognosis in lung cancer is poorly understood, which could lead to population bias in prospective studies. We retrospectively analyzed 1011 lung cancer patients whose tumor samples were successfully profiled by high-throughput sequencing panel including GI/HRD score. Alterations of many cancer suppressor genes were associated with higher GI/HRD scores, biallelic inactivation of *TP53* was correlated with a high GI/HRD score. A combination of two gene alterations exhibited a higher GI/HRD scores than single gene alterations. The GI/HRD score was associated with advanced stages in lung adenocarcinoma but not in lung squamous cell carcinoma. Furthermore, patients with higher GI/HRD scores had significantly shorter overall survival and progression-free survival than patients with lower GI/HRD scores. Finally, patients with a combination of a higher GI/HRD scores and *TP53* alteration exhibited an extremely poor prognosis compared with patients with a lower GI/HRD scores and wild-type *TP53* (overall survival, training cohort, hazard ratio (HR) = 8.56, *P* < 0.001; validation cohort, HR = 6.47, *P* < 0.001; progression-free survival, HR = 4.76, *P* < 0.001). Our study revealed the prognostic value of the GI/HRD score in lung adenocarcinoma, but not for all lung cancer. Moreover, the combination of the GI/HRD score and *TP53* status could be a promising strategy to predict the prognosis of patients with lung adenocarcinoma.

## Introduction

Lung cancer is still the leading cause of cancer death worldwide^1^. Non-small cell lung cancer (NSCLC) is the most common subtype. Although chromosome disruption is not associated with survival, dynamic chromosomal instability is an independent risk variable for recurrence or death^2^, which indicates that different features of genomic instability exhibit distinct prognostic value. Homologous recombination repair (HRR) is an error-free form for restoring double-strand DNA breaks. The homologous recombination deficiency (HRD) is mainly applied in cancers with genetic and epigenetic inactivation of homologous recombination components^3^. Other than directly testing mutations and promoter methylation of *BRCA1/2* and other HRR-related genes, there are several methods to test the “effects” of HRD, including the SNP-based HRD score^4^, single base substitution-based signature 3 (see ref. ^5^) and mutational signature-based HRDetect^6^. The HRD score is also known as genomic instability (GI) score, which approved by the U.S. Food and Drug Administration as a companion diagnostic for niraparib and olaparib in ovarian cancer and is broadly used in prospective clinical trials^7,8^.

The GI/HRD score was obtained by adding LOH, LST, and TAI together. Briefly, LOH was defined as the number of homozygous segments with zero copies of minor alleles that were longer than 15 Mb and shorter than the whole chromosome. TAI was defined as the number of sub-chromosomal allelic imbalanced regions with two alleles having uneven copy numbers, which extend to sub-telomere, do not cross the centromere and of longer than 11 Mb. The LST was defined as the number of break points between regions longer than 10 Mb after exclusion of regions shorter than 3 Mb. The GI/HRD score is not only related to PARP inhibitors and platinum-containing chemotherapy but also could be potential biomarkers in cancer immunotherapy^9,10^. In addition, the progression-free survival is associated with the GI/HRD score in several cancer types in The Cancer Genome Atlas (TCGA) dataset^11^. There is a strong positive correlation between the mean GI/HRD score per cancer type and its *TP53* mutation ratio in pan-cancer analysis^12^. Revealing the association between the GI/HRD score and clinicopathological characteristics in lung cancer is a promising area of study, which could provide an opportunity for personalized treatment.

According to pan-cancer analysis of the TCGA project, the GI/HRD score is higher in lung squamous cell carcinoma (LUSC) and lung adenocarcinoma (LUAD) than in most other tumors^13^. HRD-associated mutational signatures is related to good response of PARP inhibitor and platinum-based therapy^14^. The GI/HRD score is also associated with an enhanced neoadjuvant immunotherapy response in NSCLC^10^, but there is no sufficient level of efficacy for single agent talazoparib in LUSC patients with alterations of HRR genes in a phase 2 clinical study (S1400G)^15^. However, the potential predictive value of the GI/HRD score for prognosis and the clinical available genomic instability risk model are still lacking in NSCLC. Therefore, we conducted this large-scale real-world observational study to investigate the clinicopathological characteristics and prognosis of NSCLC patients with higher GI/HRD scores. Furthermore, we reported the prognostic value of the combination of GI/HRD score and *TP53* status.

## Results

### Association between the GI/HRD score and genomic alterations in NSCLC

**Fig. 1:**
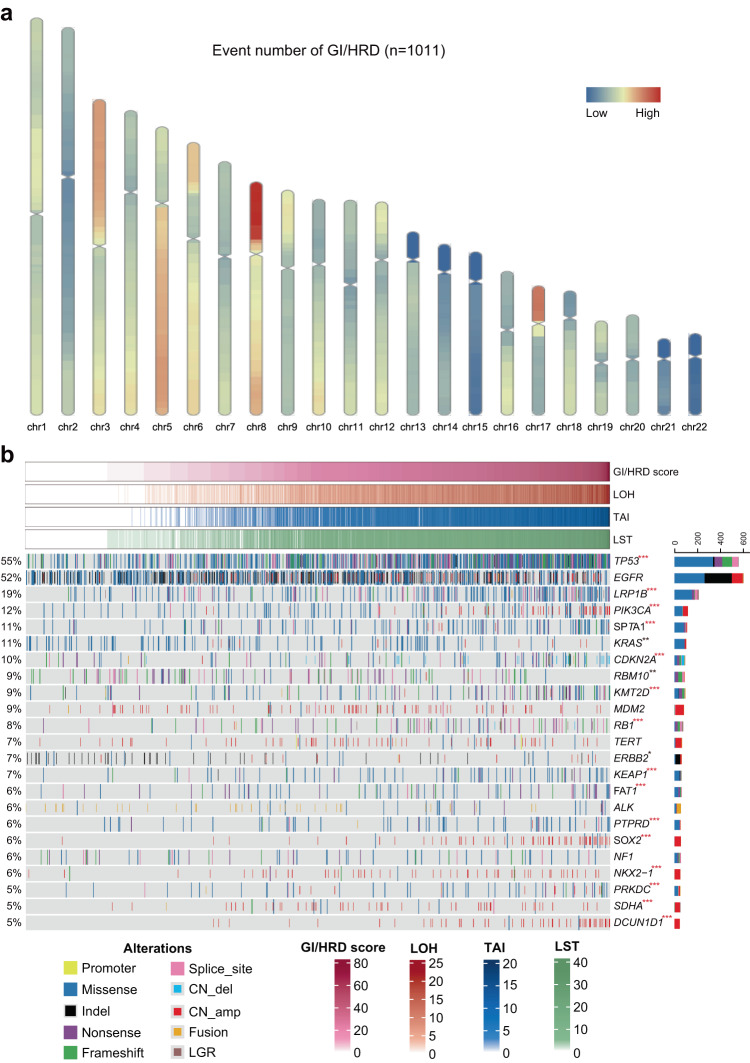
The association of GI/HRD scores and genomic alterations in NSCLC. **a** Distribution of HRD-related events (LOH, LST and TAI) on chromosomes. **b** Heatmap of genomic alterations (rate≥5%) and HRD scores. Red asterisks indicate positive correlations between gene alterations and HRD scores, and black asterisks indicate negative correlations. *Adjusted *P* < 0.05; **adjusted *P* < 0.01; *** adjusted *P* < 0.001. *n* = 1011. Student’s *t* test.

To study the relationship between GI/HRD scores and clinicopathological and genetic characteristics in NSCLC, we approved 520 genes NGS assay including over 9000 single-nucleotide polymorphisms (SNPs) distributed across the human genome in one high-throughput sequencing test. We first analyzed the chromosomal distribution of GI/HRD-related events, including loss of heterozygosity (LOH), large-scale state transition (LST) and telomere allele imbalance (TAI). LOHs were enriched on chromosomes 17p, 8p, 3p and 5q; LSTs were enriched on chromosomes 17p, 8, 5, and 3; and TAIs were enriched on chromosomes 8, 6p, 5q and 3q (Supplementary Fig. 1a). Overall, GI/HRD-related events were enriched on chromosomes 8, 17p, 5q and 3p (Fig. 1a). As GI/HRD score was sum of LOH/LST/TAI events, we performed lineal relationship analysis, which confirmed the strong correlation between GI/HRD score and LOH/LST/TAI events (Supplementary Fig. 1b). The relationship between gene alterations and GI/HRD score was shown by heatmap (Fig. 1b). Alterations of several cancer suppressor genes (including *TP53*, *LRP1B* and *CDKN2A*) were related to higher GI/HRD scores, but alterations in oncogenes (including *KRAS* and *ERBB2*) were related to lower GI/HRD scores (Fig. 1b and Supplementary Fig. 1c). These data indicate that GI/HRD-related events are enriched in specific genomic regions and inactivation of cancer suppressor genes could be the driver event of genomic instability.

*TP53* alterations were reported to be associated with higher GI/HRD scores across cancers in the TCGA dataset^11^, including prostate cancer^16,17^, endometrial cancers^18^ and ovarian cancer^19^. As one of the most common mutations in NSCLC, *TP53* alteration exhibited prognostic value in first-line tyrosine kinase inhibitor therapy^20^, postoperative adjuvant therapy^21^ and PD-1 blockade immunotherapy^22^. Recently, it was reported that biallelic alterations of *TP53* led to complex chromosome abnormalities and rapid progression in myelodysplastic neoplasms, which was defined as a unique subtype in the WHO classification^23^. However, the relationship between *TP53* alteration and GI/HRD score in NSCLC need further elucidation. Interestingly, the GI/HRD score was not elevated in patients with monoallelic *TP53* alterations, but significantly increased in patients with biallelic *TP53* alterations (Supplementary Fig. 1d). We further analyzed the percentage of biallelic *TP53* alterations in different mutational types and exons. There were diverse percentages of biallelic *TP53* alterations in patients with a single *TP53* mutation (Supplementary Fig. 1e, f). These data indicate that biallelic inactivation of *TP53*, not just mutation of *TP53*, is correlated with a high GI/HRD score.

**Fig. 2:**
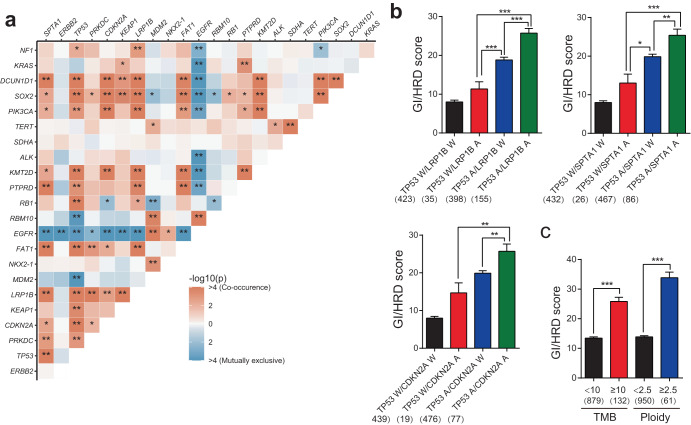
The combination of altered genes is related to the GI/HRD score in NSCLC. **a** Co-occurrence (red square) and mutually exclusive (blue square) gene alterations of NSCLC. *Adjusted *P* < 0.05; **adjusted *P* < 0.01. *n* = 1011. Fisher’s test. **b** GI/HRD score in patients with the indicated co-occurring gene alterations compared to single gene alterations. A indicates alteration, and W indicates wild-type. **c** GI/HRD score in patients with different TMB (tumor mutational burden) and ploidy levels. *P < 0.05; **P < 0.01. *n* = 1011. Student’s *t* test. Error bars represent standard error of the mean (SEM).

To further elucidate the relationship between multiple alterations of different genes, we first analyzed the correlation between alterations of different genes. Alterations of *EGFR* were mutually exclusive with many gene alterations, which indicated that mutations of *EGFR* were the strongest driving events for NSCLC. Alterations of several genes co-occurred, indicating the synergism of these genes (Fig. 2a). Next, we compared the GI/HRD score in different combinations of co-occurring altered genes (top four cancer suppressor genes that were positively related to the GI/HRD score). The GI/HRD score was higher in patients with combinations of two gene alterations than in patients with one gene alteration (Fig. 2b and Supplementary Fig. 2). Furthermore, the GI/HRD score was elevated in patients with high TMB and ploidy (Fig. 2c). With these data, we demonstrate that the GI/HRD score is associated with multiple cancer suppressor genes’ alterations, which could be the drivers of GI/HRD-related events.

### Predictive value of GI/HRD score in TNM stage of LUAD

**Fig. 3:**
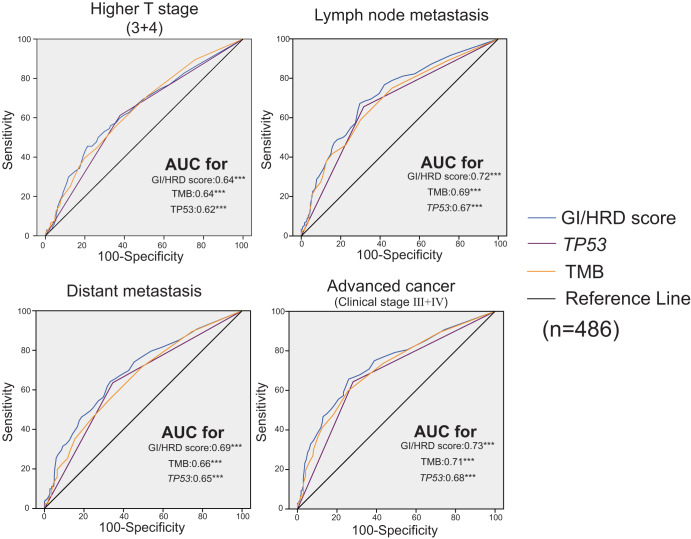
ROC curves of the prediction of characteristics of advanced cancer with GI/HRD score and other markers in LUAD with complete clinicopathological features. The area under the ROC curve and *P* value of the indicated markers are shown. ***Adjusted *P* < 0.001.

To further illustrate the clinical value of the GI/HRD score in NSCLC, we analyzed the relationship between clinicopathological characters and the GI/HRD score (Supplementary Fig. 3a). A Higher GI/HRD score was associated with male sex, older age, smoking, squamous cell carcinoma, advanced stage (including T stage, N stage and M stage) and a higher percentage of Ki67 (Supplementary Fig. 3b). As the GI/HRD score was higher in LUSC than in LUAD, which was consistent with the TCGA cohort^24^, we performed subgroup analyses in LUSC and LUAD. In our LUSC cohort and the TCGA-LUSC cohort, there were no correlations between clinicopathological characteristics and GI/HRD scores (Supplementary Figs. 3c and 4a). However, the relationship between clinicopathological characteristics and GI/HRD score was significant in our LUAD cohort but not in the TCGA-LUAD cohort (Supplementary Figs. 3d and 4a). As mutations of *EGFR* were the most common driving events for LUAD tumorigenesis, we performed correlation analysis in LUAD with *EGFR* alteration and wild-type LUAD. The GI/HRD score was related to advanced clinicopathological characteristics regardless of *EGFR* status (Supplementary Fig. 3e). In LUAD patients with complete information on TNM stage (*n* = 486), we compared the sensitivity and specificity of the GI/HRD score with other genomic alterations for the prediction of higher T stage (T3 and T4), lymph node metastasis, distant metastasis and higher clinical stage (III and IV) through ROC curves. The GI/HRD score was a better predictor than other genomic alterations, especially for advanced clinical stage (Fig. 3). Our data reveals the value of GI/HRD score in predicting the TNM stage of LUAD.

### Identification of prognostic value of the GI/HRD score in LUAD

**Fig. 4:**
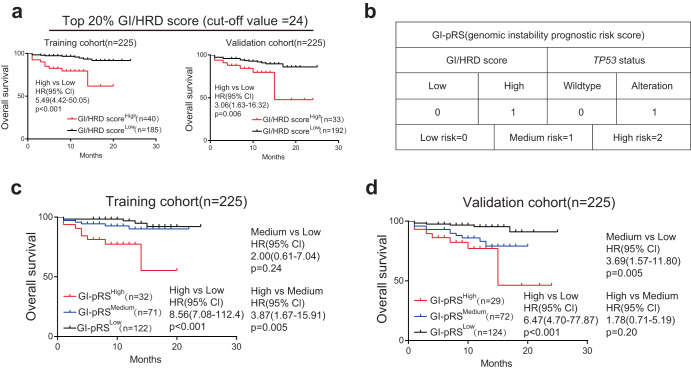
Combination of GI/HRD score and *TP53* status to predict overall and progression-free survival in LUAD. **a** Overall survival analysis of LUAD patients with high GI/HRD score versus those with low GI/HRD score in the training cohort (*n* = 225) and validation cohort (*n* = 225). The cutoff value was determined by the highest 20% (All LUAD patients in Supplementary Fig. 3D, *n* = 800). GI/HRD score ≥24 was defined as GI/HRD score^high^ for further analysis. **b** Model of the genomic instability prognostic risk score (GI-pRS) according to multivariable analysis in Table 1. **c**, **d** Overall survival analysis was performed in the training cohort and validation cohort stratified by GI-pRS.

As the GI/HRD score was associated with TNM stage, we assumed that the GI/HRD score was related to prognosis in LUAD. Because the GI/HRD score was similar to tumor mutational burden (TMB), we first determined the cutoff value of the GI/HRD score according to TMB standard (top 20%^25^). For all patients with LUAD (*n* = 800), the cutoff value of the GI/HRD score for the top 20% was 24. We collected the overall survival data of patients in Fig. 3 (*n* = 450, 36 patients were lost to follow-up). We randomly divided those patients into two cohorts (training and validation cohorts). There were no significant differences in clinicopathological characteristics between the two cohorts (Supplementary Table 1). Overall survival was significantly decreased (training cohort: HR = 5.49, *P* < 0.001; validation cohort: HR = 3.06, *P* = 0.006) in patients with a GI/HRD score in the top 20% (≥24, GI/HRD score^High^) compared to those with a score in the bottom 80% (<24, GI/HRD score^Low^) (Fig. 4a). We performed univariate and multivariable analyses in the two cohorts. Higher HRD, *TP53* alteration, *CDKN2A* alteration, older age and advanced TNM stage were associated with overall survival in the univariate analysis of the two cohorts. Variables with significant correlation(p < 0.05) were involved in following multivariable analysis. In multivariable analysis, high GI/HRD score, advanced T stage and lymph node metastasis were independently associated with overall survival in the training cohort; *TP53* alteration, male sex and distant metastasis were independently associated with overall survival in the validation cohort (Table 1). In TCGA-LUAD cohort, although the difference was not significant, patients with high GI/HRD scores also exhibited worse prognoses (Supplementary Fig. 4b). Patients with high GI/HRD scores also exhibited worse prognoses in the subgroup with *EGFR* alterations but not in wild-type *EGFR* subgroup in our cohorts and TCGA-LUAD cohort (Supplementary Figs. 4c and 5a, b). In different subgroups of clinical stage, the prognostic value was inconsistent in three cohorts (Supplementary Figs. 4d–g and 6a–d). To further confirm the prognostic value of the GI/HRD score in LUAD, we collected the progression-free survival data of patients in Fig. 3 (*n* = 401, 85 patients were lost to follow-up). Among these patients, a higher GI/HRD score was associated with shorter progression-free survival (Supplementary Fig. 5c). Patients with higher GI/HRD scores exhibited shorter progression-free survival regardless of *EGFR* status (Supplementary Fig. 5d, e), but the differences were not significant in different subgroups of clinical stage (Supplementary Fig. 6e–h). Overall, these data indicate that the GI/HRD score could be a prognostic biomarker in LUAD, especially for patients with *EGFR* alteration.

**Table 1:** Univariate and multivariable Cox regression analysis of variables associated with overall survival in LUAD patients of Training cohort (*n* = 225) and validation cohort (*n* = 225).

Variable	Training cohort (*n* = 225)				Validation cohort (*n* = 225)			
	Univariate analysis		Multivariable analysis		Univariate analysis		Multivariable analysis	
	HR (95% CI)	*P*	HR (95% CI)	*P*	HR (95% CI)	*P*	HR (95% CI)	*P*
GI/HRD score ≥ 24	5.59 (2.31–13.52)	<0.001	2.91 (1.18-7.18)	0.021	3.07 (1.32–7.11)	0.009	\	\
TMB ≥ 10	2.0 5 (0.60–7-01)	0.251	\	\	1.72 (0.59–5.02)	0.320	\	\
TP53 alteration	3.28 (1.26–8.54)	0.015	\	\	4.98 (1.98–12.53)	0.001	2.81 (1.08–7.29)	0.034
EGFR alteration	0.31 (0.13–0.79)	0.013	\	\	0.66 (0.30–1.44)	0.295	\	\
LRP1B alteration	2.30 (0.84–6.33)	0.107	\	\	1.37 (0.47–3.99)	0.566	\	\
PIK3CA alteration	0.05 (0.00–140.66)	0.450	\	\	1.05 (0.14–7.76)	0.966	\	\
SPTA1 alteration	1.49 (0.35–6.43)	0.594	\	\	0.04 (0.00–27.07)	0.340	\	\
KRAS alteration	2.48 (0.90–6.81)	0.079	\	\	1.06 (0.32–3.56)	0.921	\	\
CDKN2A alteration	3.48 (1.16–10.45)	0.026	\	\	3.19 (1.10–9.32)	0.034	\	\
Age ≥ 60	2.94 (1.17–7.39)	0.022	\	\	2.74 (1.21–6.22)	0.016	\	\
Male sex	2.31 (0.89–6.00)	0.087	\	\	3.67 (1.53–8.80)	0.004	3.12 (1.30–7.50)	0.011
T stage ≥ 3	9.17 (3.33–25.25)	<0.001	2.91 (1.01–8.34)	0.047	2.10 (0.95–4.63)	0.067	\	\
N stage ≥ 1	37.15 (4.97–277.64)	<0.001	17.03 (2.09–138.48)	0.008	5.69 (2.38–13.63)	<0.001	\	\
M stage = 1	9.90 (3.31–29.62)	<0.001	\	\	9.93 (3.72–26.49)	<0.001	6.74 (2.46–18.49)	<0.001

### Combination of GI/HRD score and *TP53* status for prognosis prediction in LUAD

*TP53* alteration is related to genomic instability and prognosis in multiple tumors^23,26,27^, including NSCLC^28,29^. As GI/HRD score and *TP53* alteration were independent risk variables in the multivariable analysis, we combined GI/HRD score and *TP53* alteration to generate GI-pRS (genomic instability prognostic risk score, Fig. 4b) and divided LUAD patients into three categories: low risk, GI/HRD score^Low^ and without *TP53* alteration; medium risk, GI/HRD score^High^ or with *TP53* alteration; and high risk, GI/HRD score^High^ and with *TP53* alteration. GI-pRS was associated with male sex, older age, advanced stage, TMB, alteration of *LRP1B*, *SPTA1 and CDKN2A* in LUAD patients (Table 2 and Supplementary Table 2) but not in TCGA-LUAD cohort excepted alteration of *LRP1B* (Supplementary Table 3). Although there were no significant differences between the GI-pRS^Medium^ and GI-pRS^Low^ groups in the training cohort or between the GI-pRS^High^ and GI-pRS^Medium^ groups in validation cohort, overall survival was significantly shorter in the GI-pRS^High^ group than in the GI-pRS^Low^ group (training cohort: HR = 8.56, *P* < 0.001; validation cohort: HR = 6.47, *P* < 0.001; Fig. 4c, d). Furthermore, higher GI-pRS was associated with shorter progression-free survival (high vs. low: HR = 4.76, *P* < 0.001; high vs. medium: HR = 1.99, *P* = 0.018; medium vs. low: HR = 2.29, *P* = 0.002. Supplementary Fig. 7a). In subgroup of *EGFR* alteration, patients with high GI-pRS exhibited shorter overall survival and progression-free survival comparing to patients with low GI-pRS (Supplementary Fig. 7b–d). These results indicate that the combination of GI/HRD score and *TP53* status exhibit better prognostic value than GI/HRD score alone.

**Table 2:** Comparison of the clinical characteristics of different GI-pRS in LUAD patients of Fig. 3 (*n* = 486).

Characteristics	GI-pRS = 0 (*n* = 258)	GI-pRS = 1 (*n* = 160)	GI-pRS = 2 (*n* = 68)	Adjusted *P* value
Age, y, mean (95% CI)	56.0 (54.6–57.4)	58.7 (57.1–60.3)	62.8 (60.3–65.4)	<0.001
Sex, male, *n* (%)	104 (40.3)	87 (54.4)	38 (55.9)	0.014
T stage, *n* (%)				<0.001
1–2	211 (81.8)	108 (67.2)	33 (48.5)	
3–4	47 (18.2)	52 (32.5)	35 (51.5)	
N stage, *n* (%)				<0.001
0	205 (79.5)	78 (48.8)	23 (33.8)	
1–3	53 (20.5)	82 (51.2)	45 (66.2)	
M stage, *n* (%)				<0.001
0	208 (80.6)	90 (56.3)	26 (38.2)	
1	50 (19.4)	70 (43.8)	42 (61.8)	
Clinical stage, *n* (%)				<0.001
I+II	192 (74.4)	64 (40.0)	14 (20.6)	
III+IV	66 (25.6)	96 (60.0)	54 (79.4)	

## Discussion

The GI/HRD score is widely used to evaluate “the effect” of homologous recombination deficiency in the clinic. A high GI/HRD score has been reported in many types of tumors, and the proportion is significantly higher than mutations of HRR genes^13^, which indicates that GI/HRD-related events are not only induced by inactivation of HRR genes but also related to other reasons. As a DNA damage repair (DDR)-related gene, *TP53* alteration is associated with SCNA^30^, LOH^31^ and GI/HRD score^12^ across cancers. Biallelic inactivation of *TP53* is a common event in cancer^32^, and is associated with genomic instability in multiple tumors^23,31,33^. In our study, we revealed that LOHs were enriched in 17p in NSCLC, in which *TP53* was located. In patients with biallelic alterations of *TP53*, including multiple mutations and a mutation combined with LOH of wild-type *TP53*, GI/HRD score was significantly elevated compared to monoallelic alteration or wild-type of *TP53*. We further discovered the distribution of biallelic alterations of *TP53* in different types and exons. Although *TP53* mutation is reported to be related to the GI/HRD score across cancers in TCGA^12,13^, our results reveal for the first time that biallelic alterations, not monoallelic alteration of *TP53* are associated with GI/HRD score. This is consistent with a recent report about ordered pattern of genomic evolution induced by biallelic alterations of *Trp53* in mouse model of pancreatic ductal adenocarcinoma^26^.

A higher GI/HRD score is also associated with worse progression-free survival in multiple cancer types but not in TCGA-LUSC and TCGA-LUAD cohort^11^. In our LUSC cohort, the GI/HRD score was not elevated in advanced tumor patients, which was consistent with the TCGA cohort. However, a higher GI/HRD score was significantly related to worse outcomes in LUAD. Large meta-analysis of NSCLC reveal that Caucasian population exhibited worse survival comparing to Asian population^34^. Comparing to European population, East Asian LUADs has more stable genomes and the difference was much stronger in smokers^35^. African Americans also exhibits higher genomic instability comparing to European Americans^36^. Even for patients with *EGFR* mutation treated with EGFR-TKI, response rate and PFS could be inconsistent in population of China, Europe and South America^37^. We also performed the comparison of the genetic and clinical characteristics of LUAD in our cohort and TCGA cohort. Our cohort exhibited younger diagnostic age, advanced T, M and clinical stage, higher percentage alteration of *EGFR* and lower percentage alteration of *LRP1B*, *SPTA1* and *KRAS* (Supplementary Table 4). These data indicate that the prognostic value of GI/HRD could be variant in different cohorts due to diverse genetic and clinical characteristics. Considering the differences seen in TCGA-LUAD cohort and our LUAD cohort, it is meaningful to validate the prognostic value of the GI/HRD score in other cohorts of East Asian population.

For continuous variable, the cut-off value is always an important one. For GI/HRD score in ovarian and breast cancer which response to platinum-based therapy, the cut-off value is 95% sensitivity to detect those tumors with BRCA1/2 deficient^4^. For TMB in pan-cancer, highest 20% in each histology was suitable cut-off value for predicting immune checkpoint inhibitor (ICI) treatments^25^. The technology employed, the cancer type and forecasting indicator will also conduct the differences of cut-off value. In our cohort, Although GI/HRD score was higher in patients with biallelic inactivation of HRR genes, the number of cases is too small (*n* = 10) to determine the cut-off value (Supplementary Fig. 8a). Furthermore, no matter cut-off value is 30 (cut-off value for predicting biallelic inactivation of BRCA1/2 with our NGS-panel in ovarian cancer) or 42 (Myraid HRD), GI/HRD score was not associated with improved survival of patients with platinum-based chemotherapy (Supplementary Fig. 8b). Prospective study involving NSCLC patients with biallelic inactivation of HRR genes undergoing platinum-based chemotherapy may determine cut-off value for predicting clinical response.

By combining the initial event (*TP53* alteration) and the results (GI/HRD score) of genomic instability, we generated a genomic instability prognostic risk score (GI-pRS). Compared to low GI-pRS, the overall survival HR of patients with a high GI-pRS was 6.57–8.56, and the progression-free survival HR is 4.76. GI-pRS exhibited higher HR comparing *TP53* status and GI/HRD score (*TP53*: overall survival, 3.28–4.87, progression-free survival, 2.63; GI/HRD score: overall survival, 3.06–5.49, progression-free survival, 3.12). Our research not only reveals prognostic value of GI/HRD score in LUAD, but also provides a risk model which is better for predicting LUAD patients’ survival comparing GI/HRD score or *TP53* status only. Because evaluating the mutational status of oncogenes in LUAD helps to guide targeted therapy, it is economical to detect gene alterations and GI/HRD events with one high-throughput sequencing test.

Although GI-pRS is a promising method for prognosis prediction, it still lacks validation in patients from multiple centers. In addition, the follow-up time was relatively short. Long-term follow-up and an expanded sample size would be helpful to validate the effectiveness of GI-pRS. Furthermore, The GI/HRD score is mainly used for predicting the effect of PARP inhibitor treatment in ovarian cancer^7,8^, and exhibits potential value for evaluating the response to platinum-containing neoadjuvant chemotherapy in breast cancer^4,38^. Furthermore, the GI/HRD score is associated an enhanced neoadjuvant immunotherapy response in lung cancer^10^. These clinical trials indicate that patients with higher GI/HRD scores could benefit from several therapy. Although GI/HRD score and GI-pRS exhibited better prognostic value in patients with *EGFR* alterations, the predictive value of treatment efficacy was still inconclusive due to limited number of cases (Supplementary Fig. 9).

In summary, by integrated analysis of genes alterations, GI/HRD-related events, clinicopathological characteristics and survival information of 1011 NSCLC patients, we confirmed a strong relationship between the GI/HRD score and biallelic alterations of *TP53*, revealed the prognostic value of the GI/HRD score in LUAD patients and developed GI-pRS for predicting survival. Our research provides a new method for evaluating the prognosis and genomic instability of LUAD patients.

## Methods

### Patients and clinical data collection

A total of 1011 patients were included in this retrospective study, between September 2018 and February 2022 at the Institute of Pathology and Southwest Hospital. Eligibility criteria included an age of 18 years or older, histological confirmation of the diagnosis of NSCLC, and sequencing by targeted NGS for 520 cancer-related genes with HRD status. Key exclusion criteria were unknown primary cancers and other malignancies. Clinical staging was based on the 8th edition of the American Joint Committee on Cancer for lung cancer. Pathologic diagnosis was made according to the WHO classification of thoracic tumors (4th edition). Clinical and pathological characteristics were obtained by reviewing the electronic medical records and laboratory findings and summarized in Supplementary Table 5. The dates of recurrence and death were collected to evaluate overall survival (OS) and progression-free survival (PFS). OS was calculated from the initial date of pathologic diagnosis until cancer-related death or the last follow-up. PFS was measured from the day of NGS testing to the first radiographic recurrence or cancer-related death. The follow-up cutoff date was June 30, 2022.

### DNA isolation and capture-based targeted DNA sequencing

Briefly, genomic DNA was extracted from formalin-fixed, paraffin-embedded tumor tissues and whole blood according to the manufacturer’s standard protocol (Institute of Pathology, Southwest Hospital, Chongqing, People’s Republic of China). The concentration of the DNA samples was measured with the dsDNA HS assay kit (Thermo Fisher Scientific, Waltham, MA) using a Qubit Fluorometer to ensure that genomic DNA was greater than 30 ng. Then, DNA shearing was performed using Covaris M220, followed by end repair, phosphorylation, adaptor ligation and polymerase chain reaction (PCR) amplification. The purified pre-enrichment library was hybridized to an OncoScreenPlus^TM^ (Burning Rock, Guangzhou, China) panel covering 520 human cancer-related genes as well as more than 9000 single-nucleotide polymorphisms (SNPs) located throughout the genome, followed by hybrid selection with magnetic beads, and PCR amplification. The quality and size distribution of the libraries were assessed by a dsDNA HS assay kit (Thermo Fisher Scientific, Waltham, MA) using a Qubit Fluorometer and a High Sensitivity D1000 ScreenTape kit using 4200 TapeStation (Agilent Technologies, CA, USA). Indexed samples were then sequenced on a NextSeq sequencer (Illumina, San Diego, CA) with paired-end reads (read length, 150 bp) and an average sequencing depth of 1000× for tissue samples and 200× for whole blood samples.

### Sequence data analysis

Sequence data were mapped to the reference human genome (hg19) using Burrows-Wheeler Aligner version 0.7.10^39^. Local alignment optimization, duplication marking and variant calling were performed using Genome Analysis Tool Kit version 3.2^40^ and VarScan version 2.4.3^41^. Tissue samples were compared against their own white blood cell control to identify somatic variants. Variants were filtered using the VarScan fpfilter pipeline, and loci with a depth less than 100 were filtered out. Base calling in plasma and tissue samples required at least 8 supporting reads for single-nucleotide variations and 2 and 5 supporting reads for insertion and deletion variations, respectively. Variants with a population frequency over 0.1% in the ExAC, 1000 Genomes, dbSNP or ESP6500SI-V2 databases were grouped as single-nucleotide polymorphisms and excluded from further analysis. The remaining variants were annotated with ANNOVAR (2016-02-01 release)^42^ and SnpEff version 3.6^43^. Analysis of DNA translocation was performed using Factera version 1.4.3^44^. Copy number variations (CNVs) were analyzed based on the depth of coverage data of capture intervals. Coverage data were corrected against sequencing bias resulting from GC content and probe design. The average coverage of all captured regions was used to normalize the coverage of different samples to comparable scales. Copy number was calculated based on the ratio between the depth of coverage in tumor samples and average coverage of an adequate number (*n* > 50) of samples without copy number variations as references per capture interval. A CNV was called if the coverage data of the gene region was quantitatively and statistically significant from its reference control. The limit of detection for CNVs was 1.5 for copy number deletions and 2.64 for copy number amplifications.

### Tumor mutation burden (TMB) calculation

TMB per patient was computed as a ratio between the total number of nonsynonymous mutations detected and the total coding region size of the gene panel using the equation below. The mutation count included nonsynonymous SNVs and Indels detected within the coding region and ±2 bp upstream or downstream and did not include hot mutation events, CNVs, SVs, and germline SNPs. Only mutations with an allelic fraction (AF) ≥ 2% for tissue samples and ≥0.2% for plasma samples were included in the mutation count. For accurate TMB calculation, the maximum AF (maxAF) should be ≥5% for tissue samples and ≥1% for plasma samples. The total size of the coding region for estimating TMB was 1.003 Mb for the 520-gene OncoScreenPlus panel.

$${\rm{TMB}}=\frac{{\rm{mutation\; count}}({\rm{except\; for\; CNV}},{\rm{SV}},{\rm{SNPs}},{\rm{and\; hot\; mutations}})}{1.003{\rm{Mb}}}$$

### Genomic instability (GI)/homologous recombination deficiency (HRD) score

We used HRD score to estimate genomic instability. For calculating HRD score, Burning Rock Instability Detection of the GEnome (BRIDGE) algorithm was developed based on over 9000 single-nucleotide polymorphisms (SNPs) distributed across the human genome, which were also included in 520-gene NGS assays. The allele-specific copy number of the genome was estimated using a custom script based on the log coverage ratio (logR) and median coverage of over 9000 SNPs targeted by the OncoscreenPlus panel, as well as the allele frequency of heterozygous SNPs among them. Minor Allele Frequency (AF) and logR data for SNPs were jointly segmented using the Circular Binary Segmentation algorithm. A probabilistic model was developed to estimate the tumor copy number per segment, alongside assessing sample tumor purity and ploidy. The LOH, TAI and LST were calculated as previously described^3,45–47^. The GI/HRD scores were calculated as the sum of the LOH, TAI and LST scores.

### TP53 biallelic status

The *TP53* biallelic status was determined by screening for two mutation events, which included the following combinations: loss-of-heterozygosity (LOH) with a germline or somatic mutation, a germline and a somatic mutation, or two somatic mutations. The LOH of *TP53* was determined by analyzing the copy number of the chromosomal segment containing the gene. If the segment’s minor copy number is 0, the gene is considered to have LOH event.

### Statistical analysis

Statistical analyses were conducted with GraphPad Prism, SPSS 18, and R software. The clinicopathological characteristics of the patients were summarized as frequencies (percentages) or medians. Correlations between gene alterations and HRD scores were performed with the Student’s *t* test. Linear regression was used to analyze the correlation of the HRD score and the three HRD-related events (LOH, TAI, and LST). Co-occurrence and mutual exclusion of gene alterations were performed with the Fisher test. ROC curve analysis and univariate/multivariable Cox regression analysis were performed in SPSS 18. Prognostic values were assessed by survival analysis. Overall survival and progression-free survival were estimated using the Kaplan–Meier method and compared between cohorts or subgroups using a log-rank test. For all calculations, the tests were two-sided, and *P* < 0.05 was considered statistically significant. Correction for multiplicity was performed with Benjamini and Hochberg (BH) method in Figs. 1B, 2A, Table 2, Supplementary Fig. 3B–E, and Supplementary Tables 2–4.

### Compliance with ethical standards

This study was performed with the approval of the ethics committee of Southwest Hospital and was in accordance with regulations issued by the National Health Commission of China and the Helsinki Declaration, written informed consent was obtained from each subject.

### Reporting summary

Further information on research design is available in the Nature Research Reporting Summary linked to this article.

### Supplementary information

Supplementary file

REPORTING SUMMARY

## Data Availability

Phenotype and genotype data can be found here: https://ngdc.cncb.ac.cn/omix (OMIX repository, accession number OMIX002311). Any other associated data supporting the findings of this study are available from the corresponding author upon request.

## Code availability

This study utilized publicly available packages with R version 4.1.0. The code used for data analysis and figure generation presented in this publication can be obtained upon request. However, custom code for segment modeling remains proprietary, as it is part of commercial product.
