# Supplementary material for: Combination of genomic instability score and TP53 status for prognosis prediction in lung adenocarcinoma
Source: NPJ Precis Oncol. 2023 Oct 31;7:110. doi: 10.1038/s41698-023-00465-x (PMC10618567; doi:10.1038/s41698-023-00465-x)
Supplement: Supplementary file 1 — Supplementary file [file 41698_2023_465_MOESM1_ESM.pdf]

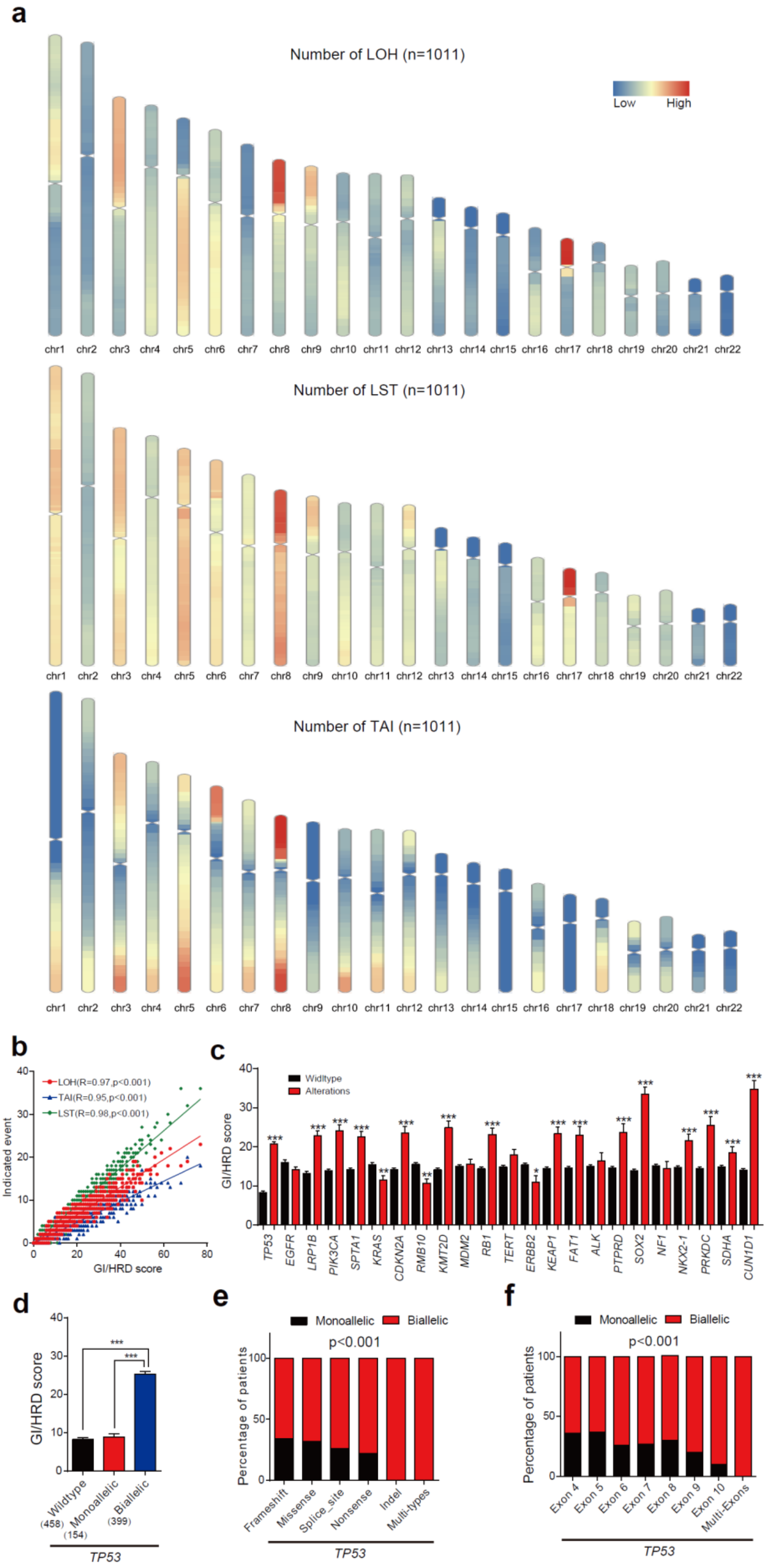
a
Number of LOH (n=1011)
Low High
chr1 chr2 chr3 chr4 chr5 chr6 chr7 chr8 chr9 chr10 chr11 chr12 chr13 chr14 chr15 chr16 chr17 chr18 chr19 chr20 chr21 chr22
Number of LST (n=1011)
chr1 chr2 chr3 chr4 chr5 chr6 chr7 chr8 chr9 chr10 chr11 chr12 chr13 chr14 chr15 chr16 chr17 chr18 chr19 chr20 chr21 chr22
Number of TAI (n=1011)
chr1 chr2 chr3 chr4 chr5 chr6 chr7 chr8 chr9 chr10 chr11 chr12 chr13 chr14 chr15 chr16 chr17 chr18 chr19 chr20 chr21 chr22
b
LOH(R=0.97,p<0.001)
TAI(R=0.95,p<0.001)
LST(R=0.98,p<0.001)
Indicated event
GI/HRD score
c
Widltype
Alterations
GI/HRD score
TP53 EGFR LRP1B PIK3CA SPTA1 KRAS CDKN2A RMB10 KMT2D MDM2 RB1 TERT ERBB2 KEAP1 FAT1 ALK PTPRD SOX2 NF1 NKX2-1 PRKDC SDHA CUN1D1
d
GI/HRD score
Widtype (458)
Monoallelic (154)
Biallelic (399)
TP53
e
Monoallelic Biallelic
p<0.001
Percentage of patients
Frameshift Missense Splice_site Nonsense Indel Multi-types
TP53
f
Monoallelic Biallelic
p<0.001
Percentage of patients
Exon 4 Exon 5 Exon 6 Exon 7 Exon 8 Exon 9 Exon 10 Multi-Exons
TP53

Supplemental Figure 1. The association of GI/HRD scores and genomic alterations in NSCLC. (a) Distribution of LOH, LST, and TAI on chromosomes. (b) Correlation analysis of the GI/HRD score and three GI/HRD-related scores (LOH, TAI and LST). n=1011, $p<0.001$, Pearson's r test. (c) GI/HRD scores in patients with the indicated gene alterations compared to wild-type patients. *adjusted $p<0.05$; **adjusted $p<0.01$; *** adjusted $p<0.001$. n=1011. Student's t test. (d) Analysis of GI/HRD scores in patients with wild-type, monoallelic inactivation and biallelic inactivation of *TP53.* wild-type, n=458; monoallelic inactivation, n=154; biallelic inactivation, n=399. ***$p<0.001$. n=1011. student's t test. (e, f) Distribution of monoallelic inactivation and biallelic inactivation in indicated *TP53* mutational types and exons. Fisher's exact test. Error bars represent standard error of the mean (SEM).

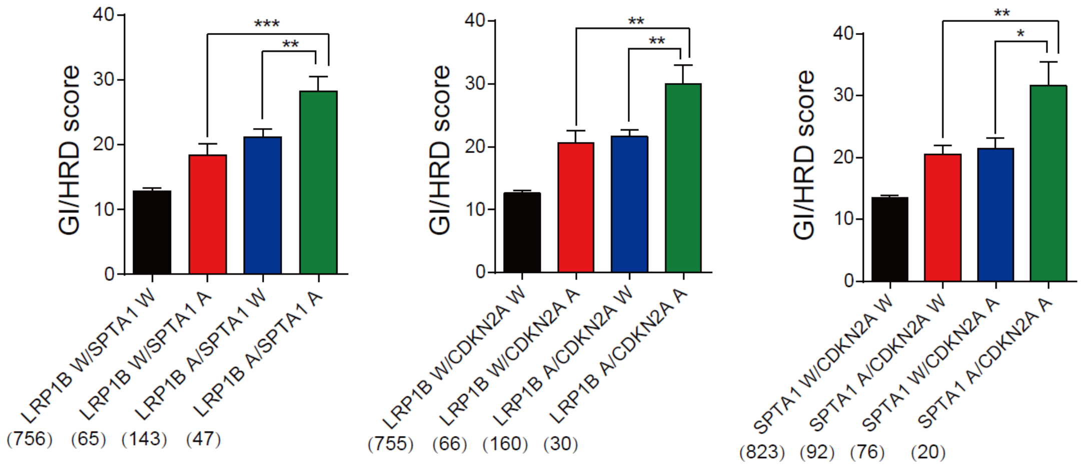

Supplemental Figure 2. GI/HRD score in patients with the indicated co-occurring genes alterations compared to single gene alterations. A indicates alteration, and W indicates wild-type. Error bars represent standard error of the mean (SEM).

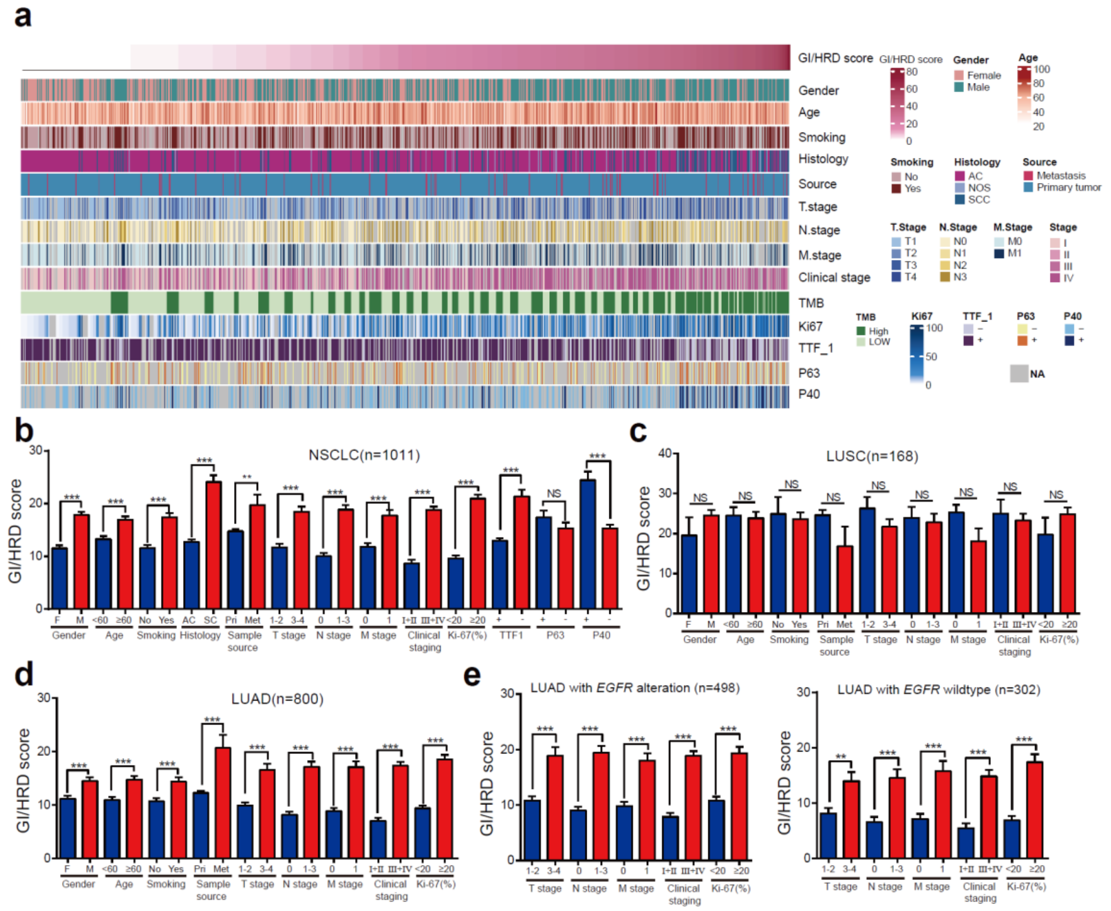

Supplemental Figure 3. The association of GI/HRD scores and Clinical Characteristics in NSCLC. (a) Landscape of clinicopathological characteristics sorted by GI/HRD score. (b) Distribution of GI/HRD scores in the indicated clinicopathological characteristic subgroups of NSCLC. (c, d) Analysis of the correlation of the GI/HRD score with the indicated clinicopathological characteristics in LUSC and LUAD. (e) GI/HRD score in patients grouped by various clinicopathological features in LUAD with/without *EGFR* alteration. Error bars represent standard error of the mean (SEM).

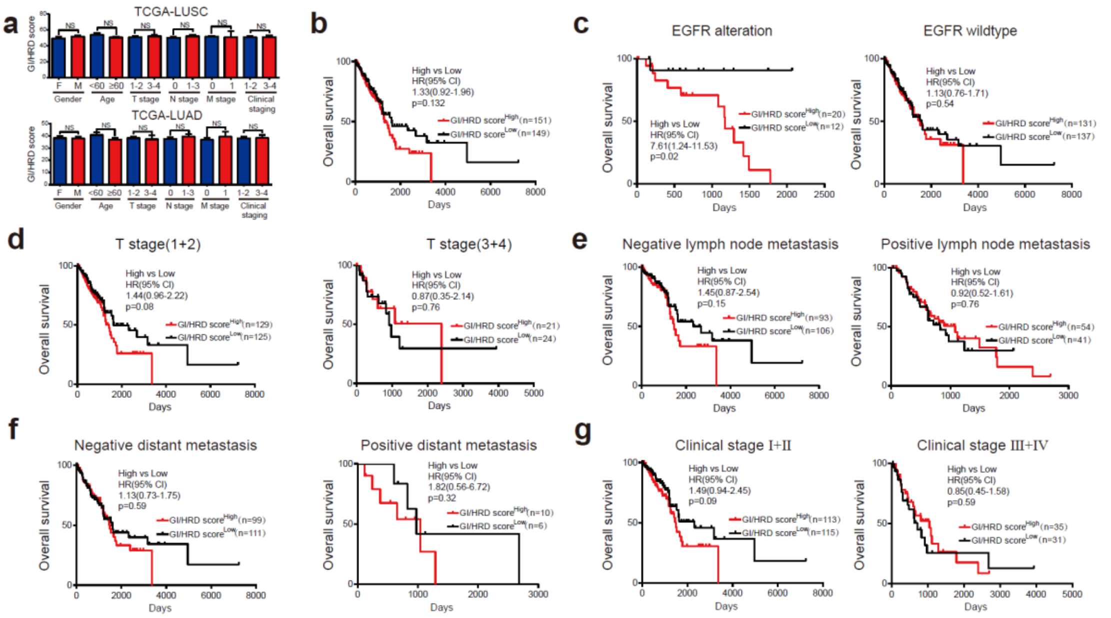

Supplemental Figure 4. Prognostic analysis of GI/HRD score in TCGA-LUAD cohort. (a) Analysis of the correlation of the GI/HRD score with the indicated clinicopathological characteristics in LUSC and LUAD in the TCGA cohort. Student's t test. (b) Overall survival analysis of TCGA-LUAD patients with high GI/HRD score versus those with low GI/HRD score. The cutoff value was determined by median value according to previous report[1]. n=300. (c-g) Kaplan–Meier curves of overall survival of TCGA-LUAD patients stratified by GI/HRD score in the indicated subgroups. Log-rank test for survival analysis. Error bars represent standard error of the mean (SEM).

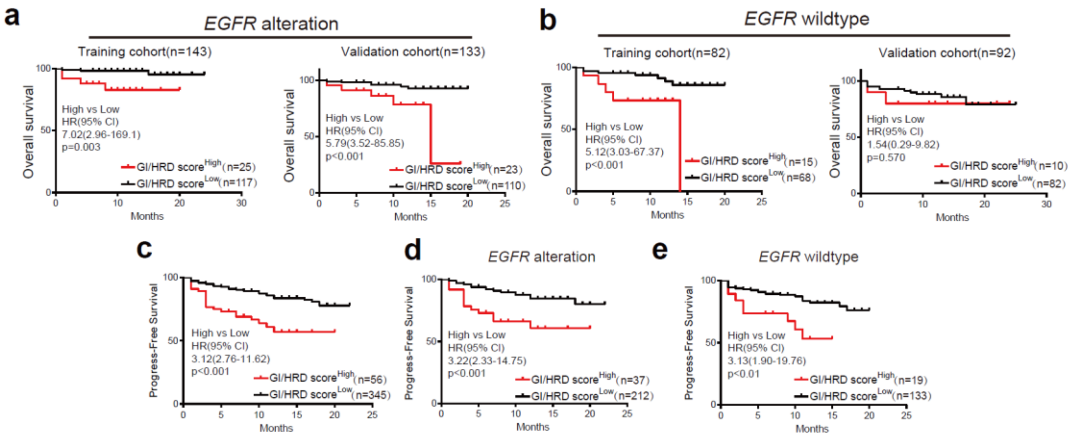

Supplemental Figure 5. Prognostic value of GI/HRD score in subgroup with different EGFR

status. (a, b) Overall survival analysis of LUAD patients stratified by GI/HRD score in subgroup of *EGFR* alteration and wild-type *EGFR*. (c) Progression-free survival analysis of LUAD patients with high GI/HRD scores versus those with low GI/HRD scores. n=401. (d, e) in subgroup of *EGFR* alteration and wild-type *EGFR*, Kaplan–Meier curves of progression-free survival of patients with high GI/HRD score versus those with low GI/HRD score. Log-rank test for survival analysis.

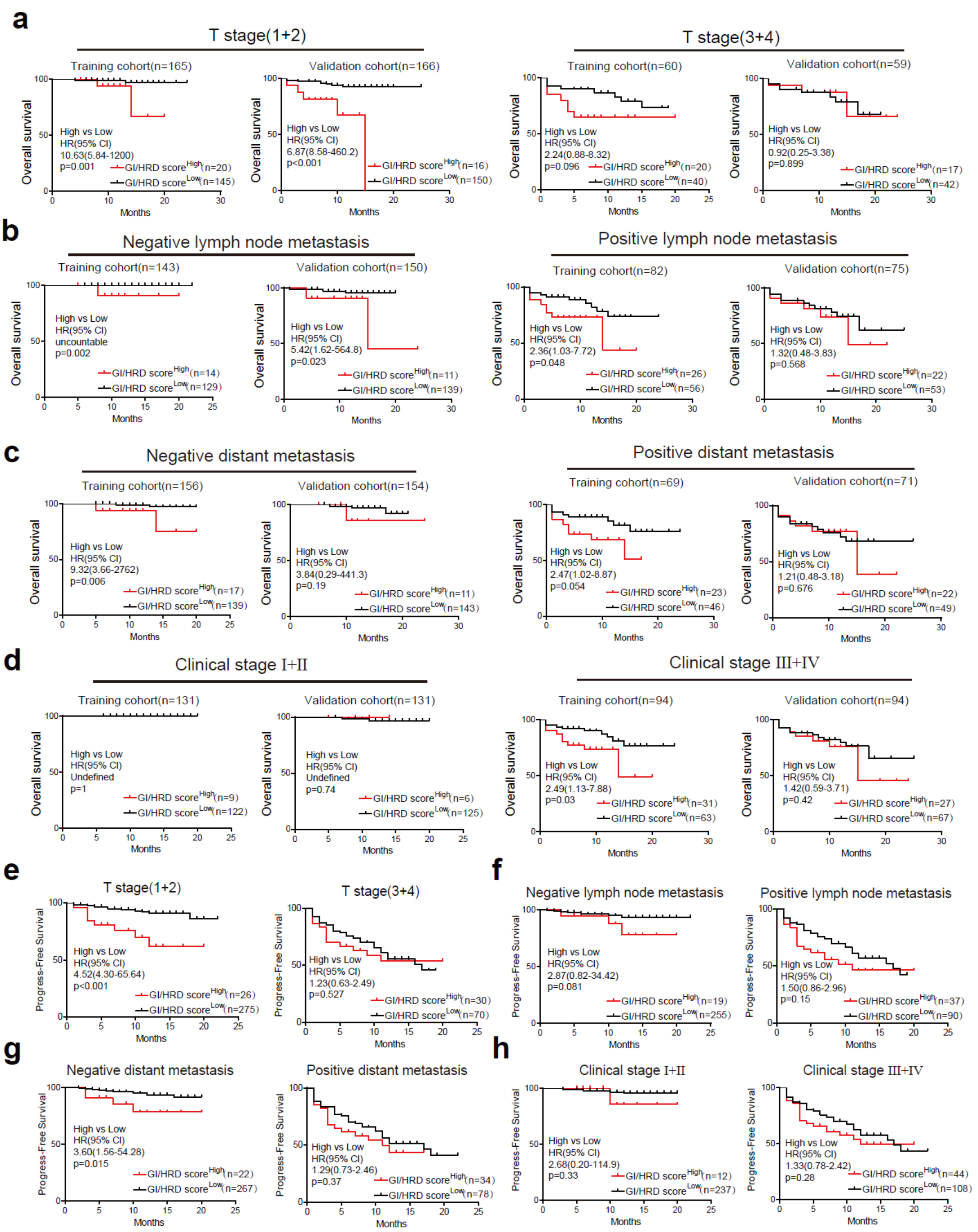

Supplemental Figure 6. Prognostic value of GI/HRD score in subgroup with different TNM status. (a-d) Overall survival analysis of LUAD patients with high GI/HRD score versus those with low GI/HRD score in the indicated subgroups in the training and validation cohorts. (e-h) Kaplan–Meier curves of progression-free survival of LUAD patients stratified by GI/HRD score in the indicated subgroups. Log-rank test for survival analysis.

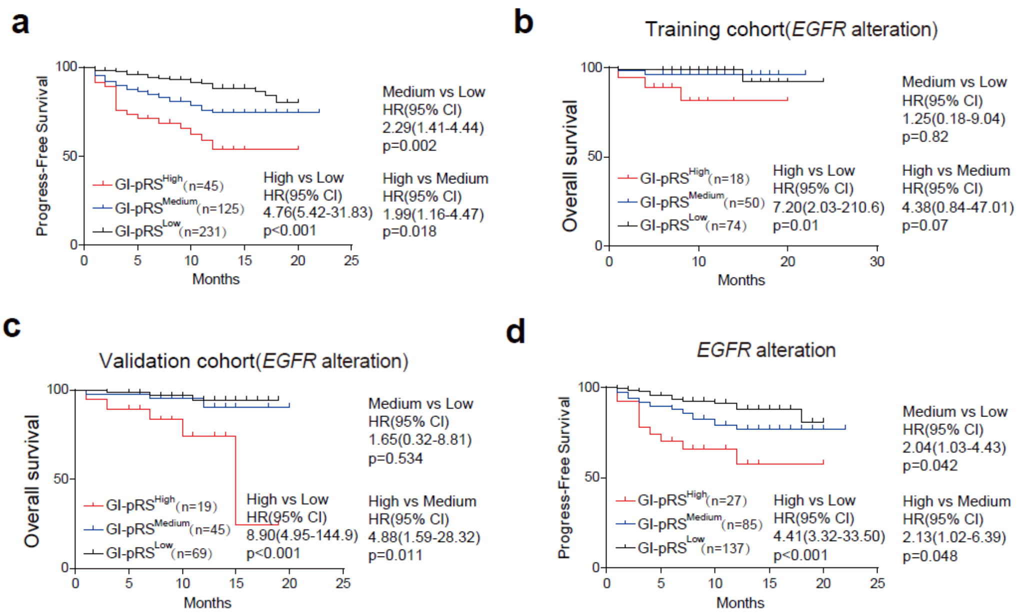

Supplemental Figure 7. Prognostic value of GI-pRS in subgroup with different EGFR status. (a) Kaplan-Meier curves of progression-free survival of LUAD patients stratified by GI-pRS. n=401. (b, c) Overall survival analysis of two cohorts stratified by GI-pRS in subgroup of *EGFR* alterations. (d) Kaplan–Meier curves of progression-free survival of patients with different GI-pRS in subgroup of *EGFR* alterations. Log-rank test for survival analysis.

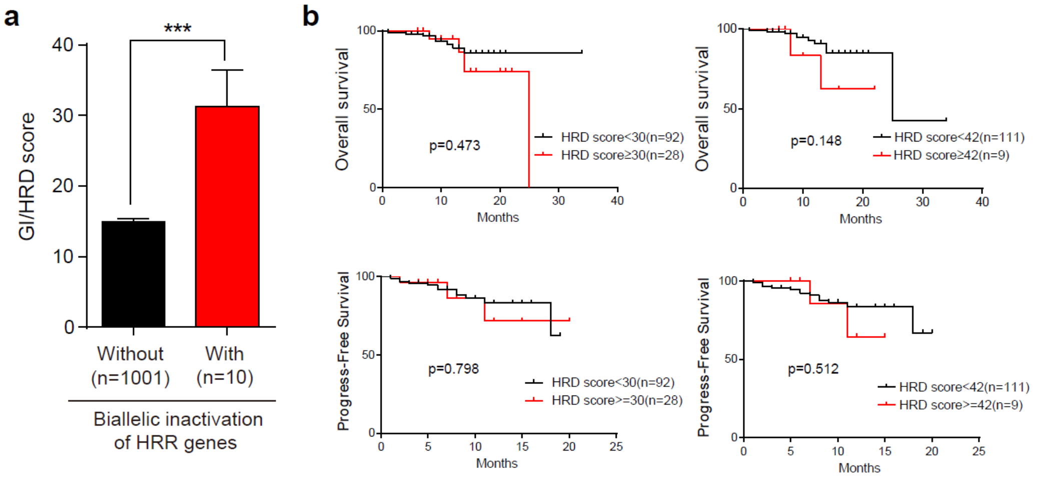

Supplemental Figure 8. The association of GI/HRD scores and inactivation of HRR genes in NSCLC. (a) Analysis of GI/HRD scores in patients with/without biallelic inactivation of HRR genes (BRCA1, BRCA2, PALB2, BARD1 and the RAD54L). ***p<0.001. n=1011.

student's t test. (b) Kaplan-Meier curves of overall and progression-free survival of LUAD patients treated with platinum-chemotherapy stratified by indicated cut-off value of GI/HRD score. n=120. Log-rank test for survival analysis. Error bars represent standard error of the mean (SEM).

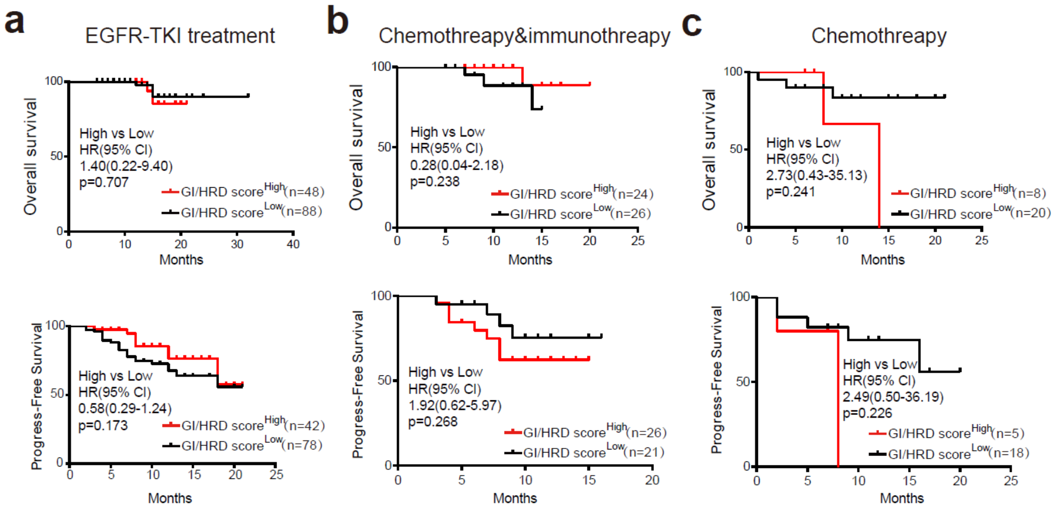

Supplemental Figure 9. Survival analysis of GI/HRD score in patients treated with indicated therapy. (a-c) Overall survival and progression-free survival analysis of LUAD patients stratified by GI/HRD score in the indicated treatment subgroups. Log-rank test for survival analysis.

Supplementary Table 1 Comparison of the Genetic and Clinical Characteristics in the Training and Validation Cohorts

Characteristics	Training Cohort (n=225)	Validation Cohort (n=225)	p Value
Age, y, mean (95% CI)	58.2(56.7-59.7)	57.5(56.1-59.0)	0.503
Sex, male, n (%)	116(51.6)	96(42.7)	0.059
T stage, n (%)			0.915
1-2	165(73.3)	166(73.8)	
3-4	60(26.7)	59(26.2)	
N stage, n (%)			0.489
0	143(63.6)	150(66.7)	
1-3	82(36.4)	75(33.3)	
M stage, n (%)			0.839
0	156(69.3)	154(68.4)	
1	69(30.7)	71(31.6)	
HRD score, n (%)			0.371
≥24	40(17.8)	33(14.7)	
<24	185(82.2)	192(85.3)	
TMB, n (%)			0.517
≥10 muts/Mb	19(8.4)	23(10.2)	
<10 muts/Mb	206(91.6)	202(89.8)	
TP53 status, n (%)			0.849
Alteration	95(42.2)	97(43.1)	
Wildtype	130(57.8)	128(56.9)	
EGFR status, n (%)			0.384
Alteration	142(63.1)	133(59.1)	
Wildtype	83(36.9)	92(40.9)	
LRP1B status, n (%)			0.887
Alteration	29(12.9)	28(12.4)	
Wildtype	196(87.1)	197(87.6)	
PIK3CA status, n (%)			0.304
Alteration	15(6.7)	10(4.4)	
Wildtype	210(93.3)	215(95.6)	
SPTA1 status, n (%)			0.860
Alteration	17(7.6)	18(8.0)	
Wildtype	208(92.4)	209(92.0)	
KRAS status, n (%)			0.656
Alteration	27(12.0)	24(10.7)	
Wildtype	198(88.0)	201(89.3)	
CDKN2A status, n (%)			0.843
Alteration	13(5.8)	14(6.2)	
Wildtype	212(94.2)	211(93.8)	

Supplementary Table 2 Comparison of the genetic characteristics of different GI-pRS in LUAD patients of Figure 3(n=486)

**Characteristics**	**GI-pRS=0(n=258)**	**GI-pRS=1(n=160)**	**GI-pRS=2(n=68)**	**Adjusted p Value**
**TMB, n (%)**				**<0.001**
≥10 muts/Mb	10(3.9)	22 (13.8)	15(22.1)	
<10 muts/Mb	248(96.1)	138(86.3)	53(77.9)	
EGFR status, n (%)				0.605
Alteration	150(58.1)	106(66.3)	41(60.3)	
Wildtype	108(41.9)	54(33.8)	27(39.7)	
**LRP1B status, n (%)**				**<0.001**
Alteration	16(6.2)	29(18.1)	21(30.9)	
Wildtype	242(93.8)	131(81.9)	47(69.1)	
PIK3CA status, n (%)				0.550
Alteration	11(4.3)	12(7.5)	5(7.4)	
Wildtype	247(95.7)	148(92.5)	63(92.6)	
**SPTA1 status, n (%)**				**<0.001**
Alteration	9(3.5)	20(12.5)	12(17.6)	
Wildtype	249(96.5)	140(87.5)	56(82.4)	
KRAS status, n (%)				0.564
Alteration	30(11.6)	21(13.1)	4(5.9)	
Wildtype	228(88.4)	139(86.9)	64(94.1)	
CDKN2A status, n (%)				0.111
Alteration	10(3.9)	12(7.5)	8(11.8)	
Wildtype	248(96.1)	148(92.5)	60(88.2)	

Supplementary Table 3 Comparison of the clinical and genetic characteristics of different GI-pRS in LUAD patients of TCGA cohort (n=191)

**Characteristics**	**GI-pRS=0(n=84)**	**GI-pRS=1(n=55)**	**GI-pRS=2(n=52)**	**Adjusted p Value**
Age, y, mean (95% CI)	66.3(64.0-68.6)	65.8(63.8-67.8)	63.8(61.8-65.8)	0.271
Sex, male, n (%)	44(52.4)	31(56.4)	29(55.8)	0.962
T stage, n (%)				0.696
1-2	74(88.1)	43(78.2)	46(88.5)	
3-4	10(11.9)	12(21.8)	6(11.5)	
N stage, n (%)				0.648
0	58(69.0)	37(67.3)	29(55.8)	
1-3	26(31.0)	18(32.7)	23(44.2)	
M stage, n (%)				0.528
0	79(94.0)	54(98.2)	46(88.5)	
1	5(6.0)	1(1.8)	6(11.5)	
Clinical stage, n (%)				0.727
I+II	66(78.6)	41(74.5)	37(71.2)	
III+IV	18(21.4)	14(25.5)	15(28.8)	
EGFR status, n (%)				0.566
Alteration	6(7.1)	7(12.7)	8(15.4)	
Wildtype	78(92.9)	48(87.3)	44(84.6)	
**LRP1B status, n (%)**				**0.005**
Alteration	15(17.9)	17(30.9)	26(50.0)	
Wildtype	69(82.1)	38(69.1)	26(50.0)	
PIK3CA status, n (%)				0.657
Alteration	5(6.0)	1(1.8)	4(7.7)	
Wildtype	79(94.0)	54(98.2)	48(92.3)	
SPTA1 status, n (%)				0.150
Alteration	10(11.9)	12(21.8)	16(30.8)	
Wildtype	74(88.1)	43(78.2)	36(69.2)	
KRAS status, n (%)				0.615
Alteration	26(31.0)	16(29.1)	11(21.2)	
Wildtype	58(69.0)	39(70.9)	41(78.8)	
CDKN2A status, n (%)				0.641
Alteration	1(1.2)	3(5.5)	2(3.8)	
Wildtype	83(98.8)	52(94.5)	50(96.2)	

Supplementary Table 4 Comparison of the Genetic and Clinical Characteristics of LUAD in our cohort(n=800) and TCGA Cohort(n=305)

**Characteristics**	**Our Cohort (n=800)**	**TCGA Cohort (n=305)**	**Adjusted p Value**
**Age, y, mean (95% CI)**	59.1(58.3-59.8)	65.2(64.0-66.3)	**<0.001**
Sex, male, n (%)	381(47.6)	148(48.5)	0.840
**T stage, n (%)**			**<0.001**
1-2	374(71.2)	258(84.9)	
3-4	151(28.8)	46(15.1)	
NA	275	1	
N stage, n (%)			0.197
0	309(61.9)	201(67.2)	
1-3	190(38.1)	98(32.8)	
NA	301	6	
**M stage, n (%)**			**<0.001**
0	330(65.5)	215(93.1)	
1	174(34.5)	16(6.9)	
NA	196	74	
**Clinical stage, n (%)**			**<0.001**
I+II	273(43.4)	231(77.3)	
III+IV	356(56.6)	68(22.7)	
NA	171	6	
TP53 status, n (%)			1.000
Alteration	366(45.8)	140(45.9)	
Wildtype	434(54.2)	165(54.1)	
**EGFR status, n (%)**			**<0.001**
Alteration	498(62.3)	33(10.8)	
Wildtype	302(37.7)	272(89.2)	
**LRP1B status, n (%)**			**<0.001**
Alteration	104(13.0)	203(66.6)	
Wildtype	696(87.0)	102(33.4)	
PIK3CA status, n (%)			0.688
Alteration	51(6.4)	16(5.2)	
Wildtype	749(93.6)	289(94.8)	
**SPTA1 status, n (%)**			**<0.001**
Alteration	69(8.6)	70(23.0)	
Wildtype	731(91.4)	235(77.0)	
**KRAS status, n (%)**			**<0.001**
Alteration	95(11.9)	89(29.2)	
Wildtype	705(88.1)	216(70.8)	
CDKN2A, n (%)			0.141
Alteration	47(5.9)	10(3.3)	
Wildtype	753(94.1)	295(96.7)	

Supplementary Table 5 Baseline characteristics of patients（n=1011）

Variable	Total, n (%)
No. of patients	1011
Median age at diagnosis, y	59(22-87)
Sex	
Male	570(56)
Female	441(44)
Smoking status	
Ever	378(37)
Never	413(41)
NA	220(22)
Histology	
Adenocarcinoma	800(79)
squamous cell carcinoma	168(17)
NOS	43(4)
Sample source	
Primary	946(94)
metastatic	65(6)
T Stage	
1	287(28)
2	137(14)
3	61(6)
4	171(17)
NA	355(35)
N Stage	
0	353(35)
1	32(3)
2	110(11)
3	125(12)
NA	391(39)
M Stage	
0	410(41)
1	213(21)
NA	388(38)
Clinical Stage	
I	278(27)
II	26(3)
III	110(11)
IV	363(36)
NA	234(23)

Reference:

1. Knijnenburg, T.A., *et al.* Genomic and Molecular Landscape of DNA Damage Repair Deficiency across The Cancer Genome Atlas. *Cell Rep* **23**, 239-254 e236 (2018).
