# Supplementary material for: Combination of genomic instability score and TP53 status for prognosis prediction in lung adenocarcinoma
Source: NPJ Precis Oncol. 2023 Oct 31;7:110. doi: 10.1038/s41698-023-00465-x (PMC10618567; doi:10.1038/s41698-023-00465-x)
Supplement: Supplementary file 2 — REPORTING SUMMARY [file 41698_2023_465_MOESM2_ESM.pdf]

nature portfolio

Corresponding author(s): TAO LUO; XIUWU BIAN

Last updated by author(s): 2023/05/16

Reporting Summary

Nature Portfolio wishes to improve the reproducibility of the work that we publish. This form provides structure for consistency and transparency in reporting. For further information on Nature Portfolio policies, see our Editorial Policies and the Editorial Policy Checklist.

Statistics

For all statistical analyses, confirm that the following items are present in the figure legend, table legend, main text, or Methods section.

n/a	Confirmed	
☐	☒	The exact sample size (*n*) for each experimental group/condition, given as a discrete number and unit of measurement
☐	☒	A statement on whether measurements were taken from distinct samples or whether the same sample was measured repeatedly
☐	☒	The statistical test(s) used AND whether they are one- or two-sided<br>*Only common tests should be described solely by name; describe more complex techniques in the Methods section.*
☐	☒	A description of all covariates tested
☐	☒	A description of any assumptions or corrections, such as tests of normality and adjustment for multiple comparisons
☐	☒	A full description of the statistical parameters including central tendency (e.g. means) or other basic estimates (e.g. regression coefficient) AND variation (e.g. standard deviation) or associated estimates of uncertainty (e.g. confidence intervals)
☐	☒	For null hypothesis testing, the test statistic (e.g. *F*, *t*, *r*) with confidence intervals, effect sizes, degrees of freedom and *P* value noted<br>*Give P values as exact values whenever suitable.*
☐	☒	For Bayesian analysis, information on the choice of priors and Markov chain Monte Carlo settings
☐	☒	For hierarchical and complex designs, identification of the appropriate level for tests and full reporting of outcomes
☐	☒	Estimates of effect sizes (e.g. Cohen's *d*, Pearson's *r*), indicating how they were calculated

Our web collection on statistics for biologists contains articles on many of the points above.

Software and code

Policy information about availability of computer code

Data collection	TCGA data of lung cancer was downloaded in cbioportal and HRD score was downloaded in previous paper(PMID: 26015868)
Data analysis	TCGA data of lung cancer was downloaded in cbioportal and HRD score was downloaded in previous paper(PMID: 26015868)

For manuscripts utilizing custom algorithms or software that are central to the research but not yet described in published literature, software must be made available to editors and reviewers. We strongly encourage code deposition in a community repository (e.g. GitHub). See the Nature Portfolio guidelines for submitting code & software for further information.

Data

Policy information about availability of data

All manuscripts must include a data availability statement. This statement should provide the following information, where applicable:

- Accession codes, unique identifiers, or web links for publicly available datasets
- A description of any restrictions on data availability
- For clinical datasets or third party data, please ensure that the statement adheres to our policy

Any other associated data supporting the findings of this study are available from the corresponding author upon request.

Research involving human participants, their data, or biological material

Policy information about studies with human participants or human data. See also policy information about sex, gender (identity/presentation), and sexual orientation and race, ethnicity and racism.

Reporting on sex and gender	570 male and 441 female.
Reporting on race, ethnicity, or other socially relevant groupings	Chinese.
Population characteristics	A total of 1011 patients were included in this retrospective study, between September 2018 and February 2022 at the Institute of Pathology and Southwest Hospital.
Recruitment	Eligibility criteria included an age of 18 years or older, histological confirmation of the diagnosis of NSCLC, and sequencing by targeted NGS for 520 cancer-related genes with HRD status. Key exclusion criteria were unknown primary cancers and other malignancies. Clinical staging was based on the 8th edition of the American Joint Committee on Cancer for lung cancer. Pathologic diagnosis was made according to the WHO classification of thoracic tumors (4th edition).
Ethics oversight	This study was performed with the approval of the ethics committee of Southwest Hospital and was in accordance with regulations issued by the National Health Commission of China and the Helsinki Declaration, written informed consent was obtained from each subject.

Note that full information on the approval of the study protocol must also be provided in the manuscript.

Field-specific reporting

Please select the one below that is the best fit for your research. If you are not sure, read the appropriate sections before making your selection.

☒ Life sciences ☐ Behavioural & social sciences ☐ Ecological, evolutionary & environmental sciences

For a reference copy of the document with all sections, see nature.com/documents/nr-reporting-summary-flat.pdf

Life sciences study design

All studies must disclose on these points even when the disclosure is negative.

Sample size	1011
Data exclusions	Key exclusion criteria were unknown primary cancers and other malignancies.
Replication	A total of 1011 patients were included in this retrospective study
Randomization	yes
Blinding	yes

Reporting for specific materials, systems and methods

We require information from authors about some types of materials, experimental systems and methods used in many studies. Here, indicate whether each material, system or method listed is relevant to your study. If you are not sure if a list item applies to your research, read the appropriate section before selecting a response.

Materials & experimental systems

n/a	Involved in the study	
☒	☐	Antibodies
☒	☐	Eukaryotic cell lines
☒	☐	Palaeontology and archaeology
☒	☐	Animals and other organisms
☐	☒	Clinical data
☒	☐	Dual use research of concern
☒	☐	Plants

Methods

n/a	Involved in the study	
☒	☐	ChIP-seq
☒	☐	Flow cytometry
☒	☐	MRI-based neuroimaging

Clinical data

Policy information about clinical studies

All manuscripts should comply with the ICMJE guidelines for publication of clinical research and a completed CONSORT checklist must be included with all submissions.

Clinical trial registration	This study was performed with the approval of the ethics committee of Southwest Hospital and was in accordance with regulations issued by the National Health Commission of China and the Helsinki Declaration, written informed consent was obtained from each subject.
Study protocol	A total of 1011 patients were included in this retrospective study, between September 2018 and February 2022 at the Institute of Pathology and Southwest Hospital. Eligibility criteria included an age of 18 years or older, histological confirmation of the diagnosis of NSCLC, and sequencing by targeted NGS for 520 cancer-related genes with HRD status. Key exclusion criteria were unknown primary cancers and other malignancies. Clinical staging was based on the 8th edition of the American Joint Committee on Cancer for lung cancer. Pathologic diagnosis was made according to the WHO classification of thoracic tumors (4th edition).
Data collection	The follow-up cutoff date was June 30, 2022.
Outcomes	OS was calculated from the initial date of pathologic diagnosis until cancer-related death or the last follow-up. PFS was measured from the day of NGS testing to the first radiographic recurrence or cancer-related death.
